# Supplementary material for: Grains on the brain: A survey of dog owner purchasing habits related to grain-free dry dog foods
Source: PLoS One. 2021 May 19;16(5):e0250806. doi: 10.1371/journal.pone.0250806 (PMC8133411; doi:10.1371/journal.pone.0250806)
Supplement: S1 Table — 1 Estimated multinomial logistic regression coefficient. 2 Odds Ratio or exponentiation of the coefficient (β). 3 95% Confidence Interval of the Odds Ratio. McFadden Pseudo R-Square = 0.050. Dependent variable categories, 1 = selected ‘no grain’, 0 = did not select ‘no grain’. (DOCX) [file pone.0250806.s001.docx]

| **Variable** | **β^1^** | **Std. Error** | **P-Value** | **OR^2^** | **95% CI^3^** | |
| --- | --- | --- | --- | --- | --- | --- |
|  |  |  |  |  | **Lower Bound** | **Upper Bound** |
| **Age** |  |  |  |  |  |  |
| - 25 to 34 years | 0.047 | 0.251 | 0.850 | 1.049 | 0.641 | 1.715 |
| - 35 to 44 years | -0.095 | 0.247 | 0.699 | 0.909 | 0.560 | 1.474 |
| - 45 to 54 years | 0.086 | 0.245 | 0.726 | 1.090 | 0.674 | 1.761 |
| - 55 to 64 years | 0.173 | 0.246 | 0.482 | 1.189 | 0.734 | 1.927 |
| - 65 years or older | 0.132 | 0.271 | 0.626 | 1.141 | 0.670 | 1.943 |
| - 18 to 24 years | . | . | . | . | . | . |
| **Sex** |  |  |  |  |  |  |
| - Male | -0.359 | 0.088 | <0.0001 | 0.699 | 0.588 | 0.830 |
| - Female | . | . | . | . | . | . |
| **Country** |  |  |  |  |  |  |
| - Germany | 0.827 | 0.424 | 0.051 | 2.286 | 0.997 | 5.243 |
| - France | -0.690 | 0.575 | 0.230 | 0.501 | 0.163 | 1.547 |
| - USA | -0.320 | 0.538 | 0.552 | 0.726 | 0.253 | 2.083 |
| - Canada | 0.623 | 0.431 | 0.148 | 1.865 | 0.801 | 4.341 |
| - UK | . | . | . | . | . | . |
| **Type of Dog** |  |  |  |  |  |  |
| - Purebred | 0.027 | 0.088 | 0.762 | 1.027 | 0.864 | 1.221 |
| - Mixed breed | . | . | . | . | . | . |
| **Only get information about pet food from a veterinarian** | -0.029 | 0.292 | 0.922 | 0.972 | 0.548 | 1.722 |
| **Importance of veterinary care in terms of your dog’s overall health** |  |  |  |  |  |  |
| - 2-5 (Not important/neutral) | 0.268 | 0.347 | 0.440 | 1.307 | 0.663 | 2.578 |
| - 6-10 (Important) | . | . | . | . | . | . |
| **Age 65 plus*USA** | 0.411 | 0.245 | 0.093 | - | - | - |
| **Only get your information about pet food from a vet*Germany** | -0.445 | 0.457 | 0.330 | - | - | - |
| **Only get your information about pet food from a vet*France** | -0.658 | 0.457 | 0.150 | - | - | - |
| **Only get your information about pet food from a vet*USA** | -0.183 | 0.358 | 0.610 | - | - | - |
| **Only get your information about pet food from a vet*Canada** | -0.454 | 0.392 | 0.246 | - | - | - |
| **Importance of vet care*Germany** | -0.234 | 0.444 | 0.598 | - | - | - |
| **Importance of vet care*France** | -0.170 | 0.603 | 0.778 | - | - | - |
| **Importance of vet care*USA** | 0.549 | 0.547 | 0.315 | - | - | - |
| **Importance of vet care*Canada** | -0.463 | 0.455 | 0.309 | - | - | - |
