# Supplementary material for: Grains on the brain: A survey of dog owner purchasing habits related to grain-free dry dog foods
Source: PLoS One. 2021 May 19;16(5):e0250806. doi: 10.1371/journal.pone.0250806 (PMC8133411; doi:10.1371/journal.pone.0250806)
Supplement: S2 Table — 1 Estimated multinomial logistic regression coefficient. 2 Odds Ratio or exponentiation of the coefficient (β). 3 95% Confidence Interval of the Odds Ratio. McFadden Pseudo R-Square = 0.066. Dependent variable categories, 1 = selected ‘no grain’, 0 = did not select ‘no grain’. (DOCX) [file pone.0250806.s002.docx]

| **Variable** | **β^1^** | **Std. Error** | **P-Value** | **OR^2^** | **95% CI^3^** | |
| --- | --- | --- | --- | --- | --- | --- |
|  |  |  |  |  | **Lower Bound** | **Upper Bound** |
| **Age** |  |  |  |  |  |  |
| - 25 to 34 years | 0.056 | 0.253 | 0.826 | 1.057 | 0.644 | 1.736 |
| - 35 to 44 years | -0.097 | 0.249 | 0.696 | 0.907 | 0.556 | 1.479 |
| - 45 to 54 years | 0.038 | 0.249 | 0.880 | 1.038 | 0.638 | 1.690 |
| - 55 to 64 years | 0.119 | 0.252 | 0.637 | 1.126 | 0.687 | 1.846 |
| - 65 years or older | 0.090 | 0.278 | 0.747 | 1.094 | 0.635 | 1.885 |
| - 18 to 24 years | . | . | . | . | . | . |
| **Sex** |  |  |  |  |  |  |
| - Male | -0.292 | 0.090 | 0.001 | 0.746 | 0.626 | 0.890 |
| - Female | . | . | . | . | . | . |
| **Country** |  |  |  |  |  |  |
| - Germany | 0.601 | 0.135 | <0.0001 | 1.824 | 1.400 | 2.377 |
| - France | -0.978 | 0.177 | <0.0001 | 0.376 | 0.266 | 0.532 |
| - USA | 0.198 | 0.177 | 0.264 | 1.218 | 0.861 | 1.724 |
| - Canada | 0.135 | 0.140 | 0.333 | 1.145 | 0.871 | 1.506 |
| - UK | . | . | . | . | . | . |
| **Type of Dog** |  |  |  |  |  |  |
| - Purebred | -0.062 | 0.101 | 0.543 | 0.940 | 0.771 | 1.146 |
| - Mixed breed | . | . | . | . | . | . |
| **How many dogs do you own?** |  |  |  |  |  |  |
| - One | -0.464 | 0.210 | 0.027 | 0.628 | 0.417 | 0.948 |
| - Two | -0.023 | 0.220 | 0.918 | 0.978 | 0.635 | 1.505 |
| - Three or more | . | . | . | . | . | . |
| **How long have you owned your dog?** |  |  |  |  |  |  |
| - 0-3 years | 0.201 | 0.135 | 0.135 | 1.223 | 0.939 | 1.593 |
| - 3-6 years | 0.184 | 0.129 | 0.153 | 1.202 | 0.934 | 1.546 |
| - 6-9 years | 0.322 | 0.137 | 0.019 | 1.379 | 1.055 | 1.803 |
| - 9 or more years | . | . | . | . | . | . |
| **What was your motivation for getting a dog?** |  |  |  |  |  |  |
| - Security | 0.100 | 0.131 | 0.446 | 1.105 | 0.854 | 1.429 |
| - Companionship | 0.434 | 0.147 | 0.003 | 1.544 | 1.157 | 2.060 |
| - For your children | -0.063 | 0.127 | 0.622 | 0.939 | 0.732 | 1.205 |
| - Company for another pet | 0.253 | 0.157 | 0.107 | 1.288 | 0.947 | 1.751 |
| - Other | 0.350 | 0.157 | 0.026 | 1.419 | 1.044 | 1.930 |
| **Where did you acquire your dog from?** |  |  |  |  |  |  |
| - Pet store | -0.252 | 0.213 | 0.236 | 0.777 | 0.512 | 1.180 |
| - Animal shelter/rescue | -0.134 | 0.125 | 0.283 | 0.875 | 0.685 | 1.117 |
| - Friends/family | -0.378 | 0.141 | 0.007 | 0.685 | 0.519 | 0.904 |
| - Stray | -0.936 | 0.427 | 0.029 | 0.392 | 0.170 | 0.907 |
| - Online | 0.189 | 0.199 | 0.344 | 1.208 | 0.817 | 1.785 |
| - Other | 0.216 | 0.215 | 0.316 | 1.241 | 0.814 | 1.894 |
| - Breeder | . | . | . | . | . | . |
| **Is this your first dog?** |  |  |  |  |  |  |
| - Yes | -0.218 | 0.113 | 0.053 | 0.804 | 0.644 | 1.003 |
| - No | . | . | . | . | . | . |
| **Age 65 plus*USA** | 0.375 | 0.246 | 0.127 | - | - | - |
