# Supplementary material for: Grains on the brain: A survey of dog owner purchasing habits related to grain-free dry dog foods
Source: PLoS One. 2021 May 19;16(5):e0250806. doi: 10.1371/journal.pone.0250806 (PMC8133411; doi:10.1371/journal.pone.0250806)
Supplement: S3 Table — 1 Estimated multinomial logistic regression coefficient. 2 Odds Ratio or exponentiation of the coefficient (β). 3 95% Confidence Interval of the Odds Ratio. McFadden Pseudo R-Square = 0.061. Dependent variable categories, 1 = selected ‘no grain’, 0 = did not select ‘no grain’. (DOCX) [file pone.0250806.s003.docx]

| **Variable** | **β^1^** | **Std. Error** | **P-Value** | **OR^2^** | **95% CI^3^** | |
| --- | --- | --- | --- | --- | --- | --- |
|  |  |  |  |  | **Lower Bound** | **Upper Bound** |
| **Age** |  |  |  |  |  |  |
| - 25 to 34 years | 0.087 | 0.255 | 0.734 | 1.090 | 0.662 | 1.796 |
| - 35 to 44 years | 0.024 | 0.251 | 0.924 | 1.024 | 0.626 | 1.676 |
| - 45 to 54 years | 0.159 | 0.248 | 0.521 | 1.173 | 0.721 | 1.907 |
| - 55 to 64 years | 0.214 | 0.251 | 0.394 | 1.239 | 0.757 | 2.028 |
| - 65 years or older | 0.165 | 0.277 | 0.551 | 1.180 | 0.685 | 2.032 |
| - 18 to 24 years | . | . | . | . | . | . |
| **Sex** |  |  |  |  |  |  |
| - Male | -0.309 | 0.091 | 0.001 | 0.734 | 0.614 | 0.878 |
| - Female | . | . | . | . | . | . |
| **Country** |  |  |  |  |  |  |
| - Germany | 0.691 | 0.137 | <0.0001 | 1.997 | 1.527 | 2.612 |
| - France | -0.948 | 0.177 | <0.0001 | 0.388 | 0.274 | 0.548 |
| - USA | 0.098 | 0.180 | 0.583 | 1.103 | 0.776 | 1.569 |
| - Canada | 0.127 | 0.152 | 0.402 | 1.136 | 0.843 | 1.530 |
| - UK | . | . | . | . | . | . |
| **Type of Dog** |  |  |  |  |  |  |
| - Purebred | 0.004 | 0.090 | 0.964 | 1.004 | 0.841 | 1.199 |
| - Mixed breed | . | . | . | . | . | . |
| **Education** |  |  |  |  |  |  |
| - Less than high school | 0.063 | 0.213 | 0.769 | 1.065 | 0.701 | 1.617 |
| - High school diploma | 0.071 | 0.141 | 0.616 | 1.073 | 0.815 | 1.414 |
| - College degree | -0.097 | 0.145 | 0.504 | 0.907 | 0.682 | 1.207 |
| - Bachelor’s degree | 0.139 | 0.136 | 0.307 | 1.149 | 0.880 | 1.502 |
| - Master’s degree, PhD, Professional degree | . | . | . | . | . | . |
| **Income** |  |  |  |  |  |  |
| - First Quartile | -0.137 | 0.143 | 0.337 | 0.872 | 0.659 | 1.154 |
| - Second Quartile | -0.213 | 0.149 | 0.153 | 0.808 | 0.604 | 1.082 |
| - Third Quartile | -0.244 | 0.159 | 0.125 | 0.784 | 0.574 | 1.070 |
| - Fourth Quartile | . | . | . | . | . | . |
| **Do you have any children between the ages of 0-17 years living in your household?** |  |  |  |  |  |  |
| - Yes | -0.209 | 0.110 | 0.057 | 0.811 | 0.654 | 1.007 |
| - No | . | . | . | . | . | . |
| **Sex of the dog** |  |  |  |  |  |  |
| - Male | -0.305 | 0.091 | 0.001 | 0.737 | 0.617 | 0.881 |
| - Female | . | . | . | . | . | . |
| **How many dogs do you own?** |  |  |  |  |  |  |
| - One | -0.320 | 0.616 | 0.604 | 0.726 | 0.217 | 2.428 |
| - Two | 0.181 | 0.620 | 0.770 | 1.198 | 0.356 | 4.037 |
| - Three | 0.400 | 0.653 | 0.540 | 1.492 | 0.415 | 5.363 |
| - Four | -0.603 | 0.830 | 0.468 | 0.547 | 0.108 | 2.784 |
| - Five or more | . | . | . | . | . | . |
| **What is the size of your dog?** |  |  |  |  |  |  |
| - X-small | -0.077 | 0.343 | 0.823 | 0.926 | 0.473 | 1.813 |
| - Small | -0.252 | 0.293 | 0.389 | 0.777 | 0.438 | 1.379 |
| - Medium | -0.234 | 0.291 | 0.423 | 0.792 | 0.447 | 1.402 |
| - Large | -0.227 | 0.296 | 0.443 | 0.797 | 0.446 | 1.423 |
| - X-large | . | . | . | . | . | . |
| **What is your dog’s age?** |  |  |  |  |  |  |
| - 0-2 years | 0.149 | 0.194 | 0.443 | 1.160 | 0.794 | 1.696 |
| - 2-5 years | 0.216 | 0.151 | 0.154 | 1.241 | 0.922 | 1.670 |
| - 5-8 years | 0.185 | 0.152 | 0.223 | 1.203 | 0.894 | 1.620 |
| - 8-11 years | 0.109 | 0.163 | 0.505 | 1.115 | 0.810 | 1.534 |
| - 11 years or older | . | . | . | . | . | . |
| **Age 65 plus*USA** | 0.418 | 0.246 | 0.090 | - | - | - |
