# Supplementary material for: Grains on the brain: A survey of dog owner purchasing habits related to grain-free dry dog foods
Source: PLoS One. 2021 May 19;16(5):e0250806. doi: 10.1371/journal.pone.0250806 (PMC8133411; doi:10.1371/journal.pone.0250806)
Supplement: S4 Table — 1 Estimated multinomial logistic regression coefficient. 2 Odds Ratio or exponentiation of the coefficient (β). 3 95% Confidence Interval of the Odds Ratio. McFadden Pseudo R-Square = 0.060. Dependent variable categories, 1 = selected ‘no grain’, 0 = did not select ‘no grain’. (DOCX) [file pone.0250806.s004.docx]

| **Variable** | **β^1^** | **Std. Error** | **P-Value** | **OR^2^** | **95% CI^3^** | |
| --- | --- | --- | --- | --- | --- | --- |
|  |  |  |  |  | **Lower Bound** | **Upper Bound** |
| **Age** |  |  |  |  |  |  |
| - 25 to 34 years | 0.060 | 0.252 | 0.813 | 1.062 | 0.648 | 1.741 |
| - 35 to 44 years | -0.080 | 0.248 | 0.748 | 0.923 | 0.568 | 1.502 |
| - 45 to 54 years | 0.050 | 0.248 | 0.841 | 1.051 | 0.647 | 1.707 |
| - 55 to 64 years | 0.133 | 0.250 | 0.594 | 1.143 | 0.700 | 1.865 |
| - 65 years or older | 0.009 | 0.275 | 0.974 | 1.009 | 0.588 | 1.731 |
| - 18 to 24 years | . | . | . | . | . | . |
| **Sex** |  |  |  |  |  |  |
| - Male | -0.306 | 0.090 | 0.001 | 0.736 | 0.618 | 0.878 |
| - Female | . | . | . | . | . | . |
| **Country** |  |  |  |  |  |  |
| - Germany | -0.210 | 0.726 | 0.773 | 0.811 | 0.195 | 3.363 |
| - France | -1.848 | 0.808 | 0.022 | 0.158 | 0.032 | 0.768 |
| - USA | -0.783 | 0.707 | 0.269 | 0.457 | 0.114 | 1.829 |
| - Canada | -0.324 | 0.724 | 0.654 | 0.723 | 0.175 | 2.986 |
| - UK | . | . | . | . | . | . |
| **Type of Dog** |  |  |  |  |  |  |
| - Purebred | 0.014 | 0.089 | 0.879 | 1.014 | 0.852 | 1.207 |
| - Mixed breed | . | . | . | . | . | . |
| **Do you regularly do moderate physical exercise with your dog?** |  |  |  |  |  |  |
| - Yes | -0.154 | 0.098 | 0.119 | 0.858 | 0.707 | 1.040 |
| - No | . | . | . | . | . | . |
| **Do you believe your dog is an ideal body weight?** |  |  |  |  |  |  |
| - Yes | 0.052 | 0.133 | 0.695 | 1.054 | 0.811 | 1.369 |
| - No | . | . | . | . | . | . |
| **How much physical activity does your dog get per day?** |  |  |  |  |  |  |
| - None | -1.337 | 0.487 | 0.006 | 0.263 | 0.101 | 0.682 |
| - 0-15 minutes | -0.494 | 0.211 | 0.019 | 0.610 | 0.403 | 0.923 |
| - 15-30 minutes | -0.633 | 0.151 | <0.0001 | 0.531 | 0.395 | 0.714 |
| - 30-60 minutes | -0.384 | 0.134 | 0.004 | 0.681 | 0.523 | 0.886 |
| - 60-90 minutes | -0.339 | 0.148 | 0.023 | 0.713 | 0.533 | 0.954 |
| - More than 90 minutes | . | . | . | . | . | . |
| **How many times a day do you offer dry food (kibble) to your dog?** |  |  |  |  |  |  |
| - Once daily | -0.124 | 0.165 | 0.454 | 0.884 | 0.639 | 1.222 |
| - Twice daily | 0.044 | 0.156 | 0.776 | 1.045 | 0.770 | 1.419 |
| - Three times daily | 0.047 | 0.227 | 0.835 | 1.048 | 0.672 | 1.635 |
| - Unlimited access | . | . | . | . | . | . |
| **Other food items (dog treats, table scraps, fruits/veggies, other) given on a daily basis** |  |  |  |  |  |  |
| - One option selected | 0.138 | 0.102 | 0.173 | 1.148 | 0.941 | 1.401 |
| - Two or more options selected | 0.450 | 0.122 | <0.0001 | 1.568 | 1.234 | 1.993 |
| - No options selected | . | . | . | . | . | . |
| **Importance of nutrition in terms of your dog’s overall health** |  |  |  |  |  |  |
| - 2-5 (Not important/neutral) | -0.622 | 0.555 | 0.262 | 0.537 | 0.181 | 1.592 |
| - 6-10 (Important) | . | . | . | . | . | . |
| **Age 65 plus*USA** | 0.451 | 0.245 | 0.066 | - | - | - |
| **Importance of exercise*Canada** | 0.542 | 0.735 | 0.461 | - | - | - |
| **Importance of exercise*USA** | 1.090 | 0.710 | 0.125 | - | - | - |
| **Importance of exercise*France** | 1.020 | 0.828 | 0.218 | - | - | - |
| **Importance of exercise*Germany** | 0.709 | 0.738 | 0.337 | - | - | - |
