# Supplementary material for: Grains on the brain: A survey of dog owner purchasing habits related to grain-free dry dog foods
Source: PLoS One. 2021 May 19;16(5):e0250806. doi: 10.1371/journal.pone.0250806 (PMC8133411; doi:10.1371/journal.pone.0250806)
Supplement: S1 File — (PDF) [file pone.0250806.s005.pdf]

Consent Question

CONSENT TO PARTICIPATE IN RESEARCH

Pet food consumer habit survey

You are invited to participate in this survey conducted by faculty at the University of Guelph, Department of Animal BioSciences. The purpose of this form is to provide you with the information needed to make an informed decision about participating in this research.

The Researchers

Principal Investigator: Anna Kate Shoveller, Department of Animal BioSciences, University of Guelph, ashovell@uoguelph.ca, 519-824-4120 ext. 53140

Other Investigators: Michael von Massow, Department of Food, Agricultural and Resource Economics, University of Guelph, mvonmass@uoguelph.ca

Sydney Banton, Department of Animal BioSciences, bantons@uoguelph.ca

Julia Guazzelli Pezzali, Department of Animal BioSciences, University of Guelph, jguazzel@uoguelph.ca

If you have any questions or concerns about the research, please feel free to contact the principal investigator.

This survey is funded by The RC Hagen Company, a Canadian owned feed company.

Purpose of the Study

The purpose of this survey is to understand how dog owners across Canada, the United States and Europe choose their dog's pet food. We will be asking questions about you, your dog and the pet food that you choose to buy for your dog.The data collected from the survey will be used as part of a Master's Thesis at the University of Guelph, published in an academic journal and may be presented as an oral or poster presentation.

Procedures

If you agree to participate in this study, we would ask you to do the following things:

Completing the survey should take no more than 15 minutes, however there are no time restrictions and you make take as long as you like to complete the survey.

If you respond to this survey using a public computer, we recommend you take the following precautions upon completion of the survey to clear all private data from the computer you are using to respond to the survey:

Clear the browsing history
Clear the cache
Clear the cookies
Clear the authenticated session
LOG OFF

If you are using Internet Explorer, the first 4 steps can be accomplished by going to Tools and selecting Delete Browser History. Your browser application may have a similar system to remove potentially identifying personal information.

Risks and Benefits

There are minimal risks involved when participating in this survey, such as possibly uncomfortable questions related to income, gender and/or personal dietary/exercise habits. If at any point during the survey you do not feel comfortable answering a question, please exit the survey. The survey is anonymous and does not collect any identifying information.

By participating in this survey, you are contributing to research that will be used to improve the understanding of how dog owners make decisions when purchasing pet food.

Participation and Withdrawal

You can choose whether to be in this study or not. If you begin this survey and then choose to withdraw before completing it, you will not be compensated. The survey is anonymous and therefore not linked to your personal information. The investigator may withdraw anonymous responses from this research if circumstances arise that warrant doing so.

Rights of Research Participants

This project has been reviewed by the Research Ethics Board for compliance with federal guidelines for research involving human participants. You do not waive any legal rights by agreeing to take part in this study. If you have any questions regarding your rights and welfare as

a research participant in this study (REB 19-12-026) please contact: Director, Research Ethics, University of Guelph, reb@uoguelph.ca, 519-824-4120 ext. 56606.

Confidentiality

No individual identifiers will be attached to the survey data. Your responses on the survey will be anonymized. After the survey has concluded, the raw data collected will only be available to the researchers associated with the University of Guelph: the primary investigator and the other investigators.

Exclusion Criteria

- Relationship with researchers - no participants with a professional or personal relationship with the researchers will be allowed to participate in the research.
- Cannot be a veterinarian, dog breeder or work in the pet food industry

Inclusion Criteria

- Must be over 18 years of age
- Must own at least one dog that is consuming a nonprescription kibble diet
- Must be the primary person responsible for selecting your dog's food

Signature of Research Participant

If you have read the information provided for the study "***Pet food consumer habit survey***" as described herein, and your questions have been answered to your satisfaction, please answer the following question:

Do you agree to participate in the research outlined above?

Yes

No

Screener Question 1

What is your age?

Under 18 years

18-24 years

25-34 years

35-44 years

45-54 years

55-64 years

65 years or older

What is your biological sex?

Male

Female

Intersex

Prefer not to disclose

Screener Question 2

Screener Question 3

Screener Question 4

Which of the following pets do you currently have (if any)? Select all that apply.

Cat

Dog

Rabbit

Fish

Turtle

Hamster

Lizard

Other

Do you work in the pet food industry?

Yes

No

Are you a veterinarian or dog breeder?

Yes

No

Do you have at least one healthy dog that is consuming a non-prescription dry dog food (kibble)?

Yes

No

Who in your household is primarily responsible for choosing your dog's food?

I am primarily responsible for choosing my dog's food

I share responsibility for choosing my dog's food equally with someone else

I am not at all responsible for choosing my dog's food

Who in your household is primarily responsible for choosing your dog's food?

My husband

My wife

My partner

My father

My mother

My son

My daughter

[ ] Other

Main Survey

Which country do you live in?

France

Germany

United Kingdom

Canada

United States

Other

Including yourself, how many people live in your household?

1

2

3

4

5 or more

What is the relationship between you and your housemates?

Family

Friends

Care giver

Partner

Roommates

Other

Do you have any children between the ages of 0 and 17 living in your household?

Yes

No

What is your highest level of education?

Less than a high school diploma

High school degree or equivalent (e.g. GED, A-Levels)

College degree

Bachelor's degree (e.g. BA, BSc)

Master's degree (e.g. MA, MSc, MEd)

Professional degree (e.g. MD, DDS)

Doctorate (e.g. PhD, EdD)

What is the total combined income of your household after taxes (in Pounds)?

£0 - £24,999

£25,000 - £49,999

£50,000 - £74,999

£75,000 - £99,999

£100,000 - £124,999

£125,000 - £149,999

£150,000 or more

What is the total combined income of your household after taxes (in CAD)?

C$0 - C$24,999

C$25,000 - C$49,999

C$50,000 - C$74,999

C$75,000 - C$99,999

C$100,000 - C$124,999

C$125,000 - C$149,999

C$150,000 or more

What is the total combined income of your household after taxes (in USD)?

$0 - $24,999

$25,000 - $49,999

$50,000 - $74,999

$75,000 - $99,999

$100,000 - $124,999

$125,000 - $149,999

$150,000 or more

In a typical week, how many days do you spend more than 15 minutes vigorously exercising? Vigorous exercise is defined as any physical activity that takes hard physical effort and makes you breathe much harder than normal, such as, heavy lifting, aerobics, fast cycling, etc.

I don't vigorously exercise more than 15 minutes in a regular week

1-2 days/week

2-3 days/week

3-4 days/week

4-5 days/week

More than 5 days/week

In a typical week, how many days do you spend more than 15 minutes doing moderate exercise? Moderate exercise is defined as any physical activity that takes moderate physical effort and makes you breathe somewhat harder than normal, such as, lifting light loads, doubles tennis, cycling at a regular pace, etc. Does not include walking.

I don't moderately exercise more than 15 minutes in a regular week

1-2 days/week

2-3 days/week

3-4 days/week

4-5 days/week

More than 5 days/week

In a typical week, how many days do you spend more than 15 minutes walking? This includes at work and home and any walking you may have done for recreation, sport, exercise or to get from place to place.

I don't walk more than 15 minutes in a regular week

1-2 days/week

2-3 days/week

3-4 days/week

4-5 days/week

More than 5 days/week

In a typical week, how many days do you spend more than 15 minutes sitting? This includes at work and home, during leisure time, at a desk, reading or watching television, etc.

I don't sit for more than 15 minutes in a regular week

1-2 days/week

2-3 days/week

3-4 days/week

4-5 days/week

More than 5 days/week

If you have more than one healthy dog that is consuming a non-prescription dry dog food (kibble), please answer the following questions according to the older dog.

Do you regularly do moderate physical exercise with your dog? This includes activities that take moderate physical effort and make you breathe somewhat harder than normal, such as, hiking, jogging, rollerblading or cycling with your dog.

Yes

No

If you answered yes to the previous question, how often do you spend doing this in a typical day?

0-30 minutes

30-60 minutes

60 minutes or more

How has your exercise regimen changed after getting your dog?

Less active	No change in activity	More active

0 1 2 3 4 5 6 7 8 9 10

Which one of the following best reflects your own dietary routine? Select all that apply.

Kosher

Halal

Grain-free

Organic

Vegetarian

Vegan

Dairy-free

Raw

Ketogenic

Diabetic diet

Renal or kidney diet

Gluten-free or celiac diet

Low or carbohydrate-free diet

Low fat/ low calorie diet

Low sodium diet

Low sugar or sugar-free diet

Low fiber diet

High fiber diet

High protein diet

No processed foods (i.e. no additives, minimal ingredient diet)

Other

None of the above

Please select how strongly you agree or disagree with the following statements.

I try to eat grains as part of a healthy diet.

Strongly Disagree					Neutral					Strongly Agree
0	1	2	3	4	5	6	7	8	9	10

I try to eat more whole grains (i.e. brown rice) than refined grains (i.e. white rice)

Strongly Disagree					Neutral				Strongly Agree	
0	1	2	3	4	5	6	7	8	9	10

Data quality is very important to us. To show that you are paying attention, please select '1-2 days/week' below.

I don't sit for more than 15 minutes in a regular week

1-2 days/week

2-3 days/week

3-4 days/week

4-5 days/week

More than 5 days/week

How many dogs do you own?

1

2

3

4

5 or more

If you have more than one healthy dog that is consuming a non-prescription dry dog food (kibble), please answer the following questions according to the older dog.

What type of dog do you own?

Purebred dog

Mixed breed dog

What is the size of your dog?

X-small (up to 8 lbs or 3.6 kg)

Small (8-22 lbs or 3.6-10 kg)

Medium (22-55 lbs or 10-25 kg)

Large (55-100 lbs or 25-45.4 kg)

Giant (more than 100 lbs or 45.4 kg)

What is your dog's age?

0-2 years

2-5 years

5-8 years

8-11 years

Older than 11 years

What is the sex of your dog?

Male

Female

Neutered male

Spayed female

Do you believe that your dog is an ideal body weight?

Yes

No

Has anyone (including your veterinarian) ever told you that your dog is overweight?

Yes

No

How long have you owned your dog?

0-3 years

3-6 years

6-9 years

More than 9 years

What was your motivation for getting a dog? Select all that apply.

Security

Companionship

For your children

Company for another pet

Other

Where did you acquire your dog from?

Breeder

Pet store

Animal shelter/rescue

Friend/family

Stray

Online

Other

Is this your first dog?

Yes

No

If you answered no to the previous question, do any health conditions experienced by your previous dog influence how you feed your current dog?

Yes

No

Has your dog ever experienced any of the following conditions? Select all that apply.

Itchy skin

Hair loss

Smelly skin

Smelly stool

Soft stool

None of the above

If you selected any of the options in the previous question, did you try changing the diet to address the issue(s)?

Yes

No

Do you feed your dog a specific diet because you believe your dog has a food allergy?

Yes

No

Do you feed your dog a specific diet because your dog has been diagnosed by a veterinarian with a food allergy?

Yes

No

How much physical activity (including walks) does your dog get per day?

None

0-15 minutes

15-30 minutes

30-60 minutes

60-90 minutes

More than 90 minutes

What type of exercise does your dog typically get in a regular week? Select all that apply.

Walk

Run

Hike

Playing ball

Off leash walks

Swimming

Dog park

Agility

Other

How many times a day do you offer dry food (kibble) to your dog?

Once daily

Twice daily

Three times daily

Unlimited access

Do you give your dog any of the following items? Select all that apply.

Dog treats (daily)

Dog treats (occasionally)

Table scraps (daily)

Table scraps (occasionally)

Fruits/vegetables (daily)

Fruits/vegetables (occasionally)

Other foods (daily)

Other foods (occasionally)

None of the above

Data quality is very important to us. To show that you are paying attention, please select '30-60 minutes' below.

None

0-15 minutes

15-30 minutes

30-60 minutes

60-90 minutes

More than 90 minutes

Please answer True or False to the following questions.

I provide a fixed amount of food to my dog every day.

True

False

I restrict my dog's food intake to control weight.

True

False

I give my dog unlimited access to food each day.

True

False

I follow the feeding guidelines on my dog's pet food bag.

True

False

I feed my dog an amount of food based on my veterinarian's recommendation.

True

False

I purposely rotate my dog's dry food (kibble) to provide variety.

True

False

What type of water do you offer your dog?

Tap water (hard)

Tap water (soft)

Filtered/deionized water

Well water

Other

Where do you get your information about dog food from? Select all that apply.

Friends/family

Veterinarian

Online resources

Pet store staff

Contacting pet food companies

Breeder

Animal shelters/rescues

Online or television advertisements

Other

Where do you purchase your pet food from?

Grocery store

Veterinary clinic

Pet specialty store

Online

Other

When choosing a pet food, I look for... (select all that apply).

Size specific nutrition

Breed specific nutrition

Age specific nutrition

Digestive care

Weight control

Dental care

Joint care

Sensitive skin/stomach care

None of the above

When choosing a pet food, I look for... (select all that apply).

Poultry (chicken, turkey)

Beef

Fish

Pork

Exotic proteins (kangaroo, duck, venison, rabbit, etc)

Organic/natural

Protein #1 ingredient

Vegetarian/vegan diet

Limited ingredient diet

None of the above

When choosing a pet food, I look for... (select all that apply).

No corn

No grain

No fillers

No by-products

No wheat

No artificial colours or flavours

No soy

No pulse ingredients

Gluten-free

None of the above

Please allocate 100 points between the following items in order of importance to you when choosing a pet food (i.e. - if you believe an item is more important, assign more points to it).

Price point	0
Ingredient list	0
Claims made on the bag	0
Your pet likes it	0
Brand	0
Sustainability	0
Total	0

How important are the following items to you in terms of your dog's overall health?

Nutrition

Not important					Neutral				Extremely important	
0	1	2	3	4	5	6	7	8	9	10

Exercise

Not important					Neutral				Extremely important	
0	1	2	3	4	5	6	7	8	9	10

Regular veterinary care/vaccines

Not important					Neutral				Extremely important	
0	1	2	3	4	5	6	7	8	9	10

Socialization

Not important					Neutral					Extremely important
0	1	2	3	4	5	6	7	8	9	10

Powered by Qualtrics
